# A review on Zika vaccine development

**DOI:** 10.1093/femspd/ftad036

**Published:** 2024-01-08

**Authors:** Zhe-Yu Peng, Song Yang, Hong-Zheng Lu, Lin-Min Wang, Ni Li, Hai-Ting Zhang, Si-Yu Xing, Yi-Nan Du, Sheng-Qun Deng

**Affiliations:** The Key Laboratory of Microbiology and Parasitology of Anhui Province, the Key Laboratory of Zoon-oses of High Institutions in Anhui, Department of Pathogen Biology, School of Basic Medical Sciences, Anhui Medical University, Hefei 230032, China; Institute of Agro-products Processing, Anhui Academy of Agricultural Sciences, Hefei 230031, Anhui, China; The Key Laboratory of Microbiology and Parasitology of Anhui Province, the Key Laboratory of Zoon-oses of High Institutions in Anhui, Department of Pathogen Biology, School of Basic Medical Sciences, Anhui Medical University, Hefei 230032, China; The Key Laboratory of Microbiology and Parasitology of Anhui Province, the Key Laboratory of Zoon-oses of High Institutions in Anhui, Department of Pathogen Biology, School of Basic Medical Sciences, Anhui Medical University, Hefei 230032, China; The Key Laboratory of Microbiology and Parasitology of Anhui Province, the Key Laboratory of Zoon-oses of High Institutions in Anhui, Department of Pathogen Biology, School of Basic Medical Sciences, Anhui Medical University, Hefei 230032, China; The Key Laboratory of Microbiology and Parasitology of Anhui Province, the Key Laboratory of Zoon-oses of High Institutions in Anhui, Department of Pathogen Biology, School of Basic Medical Sciences, Anhui Medical University, Hefei 230032, China; The Key Laboratory of Microbiology and Parasitology of Anhui Province, the Key Laboratory of Zoon-oses of High Institutions in Anhui, Department of Pathogen Biology, School of Basic Medical Sciences, Anhui Medical University, Hefei 230032, China; The Key Laboratory of Microbiology and Parasitology of Anhui Province, the Key Laboratory of Zoon-oses of High Institutions in Anhui, Department of Pathogen Biology, School of Basic Medical Sciences, Anhui Medical University, Hefei 230032, China; The Key Laboratory of Microbiology and Parasitology of Anhui Province, the Key Laboratory of Zoon-oses of High Institutions in Anhui, Department of Pathogen Biology, School of Basic Medical Sciences, Anhui Medical University, Hefei 230032, China

**Keywords:** Zika virus, Mosquito-borne disease, Vaccine

## Abstract

Zika virus (ZIKV), which belongs to the Flavivirus family, is mainly transmitted via the bite of *Aedes* mosquitoes. In newborns, ZIKV infection can cause severe symptoms such as microcephaly, while in adults, it can lead to Guillain‒Barré syndrome (GBS). Due to the lack of specific therapeutic methods against ZIKV, the development of a safe and effective vaccine is extremely important. Several potential ZIKV vaccines, such as live attenuated, inactivated, nucleic acid, viral vector, and recombinant subunit vaccines, have demonstrated promising outcomes in clinical trials involving human participants. Therefore, in this review, the recent developmental progress, advantages and disadvantages of these five vaccine types are examined, and practical recommendations for future development are provided.

## Teaser

This review help readers fully understand the current progression of ZIKV vaccine and enhance the awareness of the prevention of ZIKV.

## Introduction

Zika virus (ZIKV) is a mosquito-borne flavivirus, that was isolated from a febrile rhesus monkey in the Ugandan forest (Dick et al. 1952, Kim and Shin 2022). Over the last few years, there have been multiple global outbreaks of ZIKV infections, such as the one that occurred in French Polynesia between 2013 and 2014, where 28 000 individuals were infected. Approximately 440 000 to 1 300 000 people were infected with ZIKV following the outbreak in Brazil in 2015. From 2015 to January 2017, more than 800 000 confirmed and probable cases of ZIKV infection were reported in the Americas, and millions of individuals were estimated to be affected by ZIKV (Hills et al. 2017). Currently, ZIKV infections have broken out in some places from time to time, and the scope of infections has continued to expand, posing a major threat to public health (Morabito and Graham 2017, Baker et al. 2022). For pregnant women, most ZIKV infections are asymptomatic; however, mother-to-child transmission of the virus may result in severe fetal and childhood defects such as microcephaly, lissencephaly, cortical calcifications, uveitis, and unilateral acute maculopathy. For adults, ZIKV infection may cause Guillain–Barré syndrome (GBS) (Oliveira et al. 2023, Olmo et al. 2023). Moreover, the viral RNA encodes a polypeptide precursor, and its amino terminus 1/3 produces three structural proteins, which are present in the envelope (E), premembrane/membrane (prM/M), and capsid (C) of the virion. The carboxy-terminal 2/3 of the polyprotein produces seven nonstructural proteins: NS1, NS2A, NS2B, NS3, NS4A, NS4B, and NS5 (Giraldo et al. 2023). In viral replication and polyprotein processing, the NS3 protein is crucial and contains a helicase domain at the C-terminus and a protease domain at the N-terminus (Zhou et al. 2023). Furthermore, the NS2B/NS3 protein plays a prominent role in viral replication and maintenance of protein function (Lin et al. 2022, Chen et al. 2023). The E protein contains most virus virion surfaces and plays significant roles in the virus replication cycle (Chen et al. 2023, Sun et al. 2023). The M protein, synthesized as a prM approximately 164–168 aa in length, is instrumental in the assembly and folding of the E protein (Hasan et al. 2018, Sui et al. 2023). Thus, ZIKV candidate vaccines under development use the E and prM proteins as the primary antigens (Fialho et al. 2023). In this review, we summarize several types of ZIKV vaccines under development, including live-attenuated vaccines, inactivated vaccines, nucleic acid vaccines, viral vectored vaccines, and recombinant subunit vaccines. Although these vaccines have moved from preclinical to clinical trials in various developmental stages, none have been licensed or marketed (Wang et al. 2022). In addition, several practical challenges and difficulties in developing ZIKV vaccines are discussed. For example, the influence of previous immunity against other viruses on subsequent ZIKV infection is unclear, as this immune response potentially leads to severe immune responses such as antibody-dependent enhancement (ADE). Furthermore, evaluating vaccines is challenging to evaluate vaccines when ZIKV circulation is decreasing, and vaccine efficacy cannot be estimated comprehensively because of insufficient studies on transmission.

## Inactivated vaccines

Inactivated vaccines are produced by using physical heating or chemical reagents to prevent virus particles from losing their pathogenicity and retaining their antigenicity; subsequently, the remaining virus is inserted into the body of the parasitifer (Huang et al. 2023). Inactivated vaccines can trigger potent antiviral responses against ZIKV by promoting the production of neutralizing antibodies and CD8^+^ and CD4^+^ T cells (Dutta and Langenburg 2023). To date, four inactivated ZIKV vaccines have been developed or completed phase I clinical trials (Table 1). A purified formalin-inactivated ZIKV vaccine (ZPIV) was demonstrated to protect BALB/c mice and nonhuman primates (NHPs) from fatal ZIKV infection. Subsequently, through phase I, placebo-controlled, and double-blind trials on healthy adults, ZPIV was confirmed to strongly stimulate the production of neutralizing titer antibodies. Notably, the reactions of participants who were injected with ZPIV were primarily mild, causing only minor to moderate reactogenicity with only some common symptoms, such as injection-site tenderness and headache (Modjarrad et al. 2018). A purified inactivated ZIKV vaccine (PIZV) developed by Baldwin et al. was tested for its ability to produce an immune response and protect mice from the CD-1 and AG129 strains. Mice that were administered 1.0 µg or 0.1 µg of PIZV exhibited potent immune reactions and full defense against ZIKV infection, with no observed weight loss or indications of sickness (Baldwin et al. 2018). Simultaneously, Abbink et al. conducted trials to assess the protective effects of PIZV in rhesus monkeys, the findings of which suggested that this vaccine provided good protection against ZIKV infection with high titers of neutralizing antibodies and no specific adverse clinical safety effects (Abbink et al. 2016). The PIZV has progressed to the phase 2 clinical trials, and assessed the safety and immunogenicity in healthy participants (US Centers for Disease Control and Prevention (CDC) 2023). Moreover, Kim et al. conducted experiments to assess the effectiveness of ZPIV in preventing fetal malformation due to ZIKV infection during pregnancy in C57BL/6 mice and marmoset monkeys. ZPIV significantly reduced the incidence of fetal malformation caused by ZIKV infection. Moreover, in mice, there was a positive correlation between fetal protection and the presence of neutralizing antibodies. Additionally, in marmoset monkeys, this vaccine was effective at preventing the vertical transmission of ZIKV (Kim et al. 2022). Han et al. tested the immunogenicity and safety of the TAK-426 vaccine in healthy people who were either flavivirus naive or flavivirus primed. The results showed that TAK-426 has a favorable safety profile and induces an immune response that is tolerable and acceptable (Han et al. 2021).

**Table 1.** ZIKV vaccine candidates in clinical trials.

Vaccine type	Vaccine name	Antigen	Phase	Developer(s)	Adjuvant
Inactivated vaccines	ZPIV	Whole virus	I	NIAID/WRAIR/BIDMC	Alum
	PIZV/TAK-426	Whole virus	I	Takeda Pharmaceuticals	Alum
	VLA1601	Whole virus	I	Valneva Austria GmbH	Alum
	BBV121	Whole virus	I	Bharat Biotech International	Alum
DNA vaccines	VRC5288	prM/E	I	NIAID, VRC	None
	VRC5283	prM/E	II	NIAID,VRC	None
	GLS-5700	prM/E	I	GeneOne Life Science/Inovio Pharmaceuticals	None
Live-attenuated vaccine	rZIKV/D4Δ30–713	rZIKV/D4Δ30–713	I	NIAID	None
mRNA vaccines	mRNA 1325	prM/E	II	Moderna Therapeutics	None
	mRNA 1893	prM/E	II		None
Viral vectored vaccines	MV-ZIKA-RSP	prM/E	I	Themis Bioscience GmbH	None
	MV-ZIKA	prM/E	I	Themis Bioscience GmbH	None
	ChAdOx1 ZIKA	CprME/NS	I	University of Oxford	None
	Ad26.ZIKV.001	ZIKV M-Env	I	Janssen Vaccines and Prevention B.V.	None

Abbreviations: ZPIV, ZIKV purified inactivated vaccine. PIZV, purified inactivated Zika virus vaccine. VRC, Vaccine Research Center. prM, premembrane. E, envelope. WRAIR, Walter Reed Army Institute of Research. NIAID, National Institute of Allergy and Infectious Diseases (USA). BIDMC, Beth Israel Deaconess Medical Center. ChAdOx1, Chimpanzee adenovirus Oxford 1. MV, measles virus vaccine.

In brief, inactivated vaccines have the advantage of being safe and stable during storage, and they can stimulate robust neutralizing antibody titers in healthy individuals. However, due to their low immunogenicity, inactivated vaccines often require adjuvants or multiple doses to stimulate a stronger immune response (Makhluf and Shresta 2018, Stephenson et al. 2020). Additionally, vaccine inactivation may cause epitopes to lose their effect or lead to several subneutralizing antibody reactions (Table 2) (Cimica et al. 2016).

**Table 2.** Advantages and disadvantages of different vaccine types.

Vaccine type	Advantages	Disadvantages	Principle of generation
Inactivated vaccines	High safety for immunosuppressed people. Better stability for storage.	Low immunogenicity Need adjuvants or multiple doses to enhance immunity.	Composed of viral particles along with other pathogens that were cultured.
Live-attenuated vaccines	Persistent immune response without adjuvants or multiple doses.	Not recommended for immunosuppressed people or gravidas because of potential hazards.	Reduce the virulence of a pathogen while maintaining its activity.
DNA vaccines	Better stability for storage. Better perform vaccine design by adding or deleting.	Low immunogenicity. Low therapeutic efficacy due to the degradation of DNA.	An antigen from a pathogen is cloned and inserted into the DNA plasmid.
mRNA vaccines	Provide a better safety profile because of less insertional mutations.	Low-temperature storage owing to instability. Need to boost immunization.	Synthesized with the virtually desired sequence.
Viral vectored vaccines	Induce stronger immune responses.	Not recommended for immunocompromised persons or gravidas.	Insert genes encoding the proteins of pathogenic microorganisms into the vector.

## Live-attenuated vaccines

To reduce toxicity while retaining immunogenicity and stimulating various immune responses, live-attenuated vaccines are administered to combat pathogens. There are two main approaches to developing live-attenuated vaccines: one approach involves the deliberate introduction of specific mutations into the genome of the virus, while the other approach entails the development of chimeric flaviviruses that express the prM/E proteins of ZIKV within the genetic framework of yellow fever virus (YFV) or Dengue virus (DENV) (Annamalai et al. 2017, Xie et al. 2018, Annamalai et al. 2019). The National Institute of Allergy and Infectious Diseases (NIAID) created rZIKV/D4Δ30–713, which is a mixture of viruses; used DENV-4 as its basis; and included the prM/E surface proteins of ZIKV as antigens. This creation has now progressed to the first phase of clinical testing (Table 1) (Cimica et al. 2021). A vaccine containing an NS1 protein without glycosylation was tested by Richner and colleagues on mice, who found that the vaccine could effectively guard against ZIKV infection-induced placental damage and fetal demise (Richner et al. 2017). In a separate study, Shan and colleagues developed a vaccine that had a deletion of 10 nucleotides within the 3'-untranslated region of the ZIKV genome; this vaccine exhibited high attenuation, immunogenicity, and protection in mouse models, resulting in better T-cell responses than those elicited by the original virus (Shan et al. 2017). The chimeric vaccine of ZIKV prM/E and JEV SA14-14-2 developed by the Beijing Institute of Microbiology and Epidemiology (BIME) protected mice and NHPs from ZIKV infection. This vaccine demonstrated good protection against ZIKV intrauterine dissemination in mice (Li et al. 2018). Compared to inactivated vaccines, live-attenuated vaccines generally provide fast and long-lasting immunity without requiring adjuvant or booster immunization (Pollard and Bijker 2021). However, for pregnant women and immunocompromised individuals with underlying health conditions, the live-attenuated vaccine could pose a risk to their health due to its low safety and potential hazards (Table 2) (Yeasmin et al. 2023).

## Nucleic acid vaccines

### DNA vaccines

DNA vaccines involve the replication of defective DNA from the virus. DNA vaccines use host cells to replicate endogenous viral antigens, thus inducing effective cellular and humoral immune responses (Wang et al. 2022, Xiong et al. 2022). To guard against ZIKV transmission, these vaccines typically contain the E and prM genes, which encode the E and prM proteins and are designed to elicit both types of immune responses. VRC-ZKADNA090-00-VP (VRC5283) and VRC-ZKADNA085-00-VP (VRC5288), DNA vaccines developed by NIAID, are composed of prM/E from ZIKV and a prM/E chimera, respectively. Immunogenicity studies conducted on mice and rhesus macaques showed that these vaccines could produce substantial amounts of ZIKA-specific neutralizing antibodies, with titers reaching up to 105 reciprocal EC_50_ serum dilutions. These vaccines have also demonstrated excellent protective effects against ZIKV-induced damage in the testes and sperm of rhesus macaques and mice. When injected into mice, a DNA vaccine expressing the prM/E protein was found to prevent damage to testes and sperm caused by ZIKV infection, as shown by Larocca and Tebas et al. (Larocca et al. 2016). Similarly, Abbink et al. obtained the same results in a rhesus monkey model (Abbink et al. 2016). Relevant experimental results from clinical trials showed that DNA vaccines could induce strongly neutralizing antibodies and have a reassuring safety profile (Poland et al. 2018). Subsequently, Gaudinski et al. recruited 18- to 50-year-old healthy adult participants and conducted two phase I randomized and open-label trials. The volunteers received VRC5283 and VRC5288 and their immunogenicity and adverse reactions were assessed on the seventh day after injection. These vaccines showed good safety and tolerance in trials, with local and systemic reactogenic events that tended to be mild and moderate, respectively. Most volunteers' local and systemic symptoms were pain and tenderness at the injection site and headache (Dowd et al. 2016, Gaudinski et al. 2018). Due to its favorable tolerability, VRC5283 has progressed to phase II clinical trials. However, additional clinical trials are still necessary to advance the development of this vaccine and assess its effectiveness in populations at risk (Lin et al. 2018). Another DNA candidate vaccine, GLS-5700, contained the contains plasmid pGX7201 at a 10 mg/ml concentration in sodium salt citrate buffer. In a phase I trial, Tebas et al. examined the side effects, safety, and immunogenicity of GLS-5700, and they discovered that most participants generated high levels of binding and neutralizing antibodies after three injections (Tebas et al. 2021).

DNA vaccines are stable at ambient temperature, relatively inexpensive to develop and straightforward to manufacture, making them a cost-effective and practical option for addressing epidemics in developing countries (Suschak et al. 2017, Zou et al. 2018, Yamanaka et al. 2022). However, the major issue with DNA vaccines lies in their inadequate ability to elicit an immune response in larger animals, probably because of the inconvenience of adding the vaccine amounts used in small animal systems. Moreover, unformulated and naked plasmid DNA vaccines may have low therapeutic efficacy due to the degradation of DNA (Table 2) (Hobernik and Bros 2018).

### mRNA vaccines

The operational mechanism of mRNA vaccines is akin to that of DNA vaccines, as they employ the host cell replication system to generate immunogenic proteins. Moderna and its counterparts have devised two mRNA vaccines, mRNA-1325 and mRNA-1893, which have shown encouraging results in providing comprehensive protection from ZIKV infection in AG129 mice and rhesus monkeys. Currently, mRNA-1893 are in phase II trials (Table 1) (US Centers for Disease Control and Prevention (CDC) 2023).Pardi and his team formulated an mRNA vaccine with the ZIKV prM/E gene and enveloped it with lipid nanoparticles (LNPs) to create a prM/E-mRNA-LNP vaccine. This vaccine achieved continuous immune effects after a single low-dose injection and induced a robust CD4^+^ response and the production of specific IgG antibodies targeting the E protein in C57BL/6 and BALB/c mice. Moreover, the virus challenge test showed no viral hematological symptoms in either model animal (Pardi et al. 2017). The effectiveness of humoral and cellular reactions induced by mRNA vaccines, particularly when targeting MHC-II, may be impacted by the efficiency of nucleic acid uptake by cells. In short, while the rapid production and flexibility of mRNA vaccines make them appealing options, importantly, they may pose potential risks due to insertional mutations, necessitating a focus on improving safety (Pardi et al. 2018, Rzymski et al. 2023, Wilder-Smith and Durbin 2023). Additionally, mRNA vaccines typically require LNPs for delivery, must be stored at low temperatures and are generally administered through prime-boost immunization schedules (Table 2) (Wollner and Richner 2021, Yamanaka et al. 2022).

## Viral vectored vaccines

A variety of viral vectors, including retrovirus, lentivirus, and adeno-associated virus vectors, can be utilized to create vaccines that infect host cells and prompt immune responses, including humoral and cellular components such as cytotoxic T lymphocytes (CTLs) (Zhang and Zhou 2016, Mühlebach 2017, Khoshnood et al. 2022). These vaccines are often used for genetic modifications and can provoke substantial innate and adaptive immune reactions in the host organism. In clinical trials, adenovirus and measles vectored vaccines have shown promise (Table 1) (Vemula and Mittal 2010, Capone et al. 2013, Lagunas-Rangel et al. 2017). For example, Ad26.ZIKV.001, a recombinant and replication-incompetent Ad26 vector encoding engineered ZIKV M and E proteins, was shown to elicit potent ZIKV-specific neutralizing responses with minor side effects such as fatigue and headache (Salisch et al. 2021, Shoushtari et al. 2022). Similarly, the prM/E proteins of ZIKV were expressed on a platform using the clinically approved replication-deficient chimpanzee adenovirus vector. This vaccine demonstrated nearly 100% protection against ZIKV infection and facilitated the production and maintenance of antibody and T-cell responses over the long term. Additionally, the vaccine prevented the development of viremia and the transmission of the virus in the brain and other tissues, outperforming previous vaccines in this regard (López-Camacho et al. 2018). Abbink et al. administered an adenovirus vaccine to rhesus monkeys infected with the Brazil ZIKV strain and found that neutralizing antibodies were induced in vivo, and that the virus could not be detected in the plasma (Abbink et al. 2016). Kim et al. further studied the effects of this vaccine on C57BL/6 mice and found that the neutralizing antibody could be detected by the plaque reduction neutralization test (PRNT) four weeks after vaccination. Furthermore, the vaccine could provide passive protection against ZIKV infection in juvenile pups from immunized female mice, and the degree to which IgG was transferred from the mother mice could influence the survival rate of the juvenile rat (Kim et al. 2016). The other two measles vectored vaccines, MV-ZIKA and MV-ZIKA RSP, were demonstrated to have good protective effects on infant rat fetal models, reducing the viral load of fetuses (Nürnberger et al. 2019). The formulation of viral vectored vaccines may not require the use of adjuvants, and in certain scenarios, booster immunization may be unnecessary. Studies show that even individuals or animals who have received previous measles immunization can still generate strong immune responses with measles viral vector vaccines (Abbink et al. 2018, Cox et al. 2018, Sadoff et al. 2021). However, it is recommended that viral vectored vaccines, such as those based on measles and vaccinia viruses, be given to immunocompromised individuals or pregnant women (Ura et al. 2014, Singh et al. 2019). However, while viral vectored vaccines are efficient at stimulating durable cellular and humoral immune reactions, they may also trigger an anti-adenovirus immune response that can decrease immunogenicity in subsequent vaccinations using the same viral vector (Zhang and Zhou 2016, Perdiguero et al. 2023). There may also be challenges in using the adenovirus and vaccinia viral systems for clinical purposes due to preexisting immunity toward the vectors (Table 2) (Cimica et al. 2021, Bifani et al. 2023).

## Recombinant subunit vaccines

A recombinant subunit vaccine against ZIKV is created by using plasmid DNA encoding a specific gene in bacteria, yeast, or insect cells, and this approach is considered effective and feasible for generating long-lasting protective and therapeutic immune responses (Durbin and Wilder-Smith 2017, Shukla et al. 2017, Tripathi and Shrivastava 2018, Zhou et al. 2021, Wang et al. 2022). Zhang et al. created a subunit protein vaccine that uses yeast-produced EDIII and was demonstrated to be highly efficient at producing EDIII in yeast cells while also preventing the production of cross-reactive antibodies that can enhance DENV infection (Rey et al. 2017, Zhang et al. 2019). Tai et al. designed a recombinant protein comprising residues 298–409 of ZIKV EDIII that can lead to the development of neutralizing antibodies and defend against ZIKV infection in pregnant mothers and fetuses (Tai et al. 2018). A zDIII subunit vaccine was created by Yang and colleagues using virus-like particles (VLPs) displaying zDIII. These VLPs were produced using the hepatitis B core antigen, which can produce humoral and cellular immunity against virus infection in mice. In comparison to DNA vaccines, this protein-based vaccine has the advantage of avoiding the possibility of genome insertion or related oncogenesis. Furthermore, the safety of this protein-based vaccine surpasses that of vaccines based on inactivated viruses or viral vectors, as it eliminates the risks of incomplete inactivation and adverse reactions to the vectors in the host (Yang et al. 2017). After being administered a subunit vaccine candidate that contains the ZIKV E protein and two adjuvants (namely, Co-Vaccine HTTM and alum), BALB/c, Swiss Webster, and C57BL/6 mice experienced significant increases in their IgG titers as well as in the production of neutralizing antibodies to combat ZIKV infection (To et al. 2018). Medina et al. created two different vaccine formulations by adding the Co-Vaccine HT™ and Alhydrogel® 85 adjuvants to the E protein through the S2 expression system in *Drosophila melanogaster*. Significant levels of protection were induced by both ZIKV vaccine formulations, whereby plasma obtained from cynomolgus macaques immunized with ZIKV E effectively protected mice against viral challenge and protected unborn children from the detrimental neurovirulence of ZIKV infection (Medina et al. 2018). After being given a subunit vaccine composed of the first 450 aa in the N-terminal region of the E protein (E90), immunocompetent mice were exposed to ZIKV infection during fetal development and early infancy. The offspring of vaccinated pregnant mice were protected against brain damage in addition to being completely protected against fatal ZIKV infection in the neonatal mouse model. Moreover, this vaccine is safe and has no potential risk of virulence or other adverse symptoms related to live vaccines (Han et al. 2017,, Zhu et al. 2018). Amaral et al. developed a recombinant protein vaccine utilizing poly (I:C) as an adjuvant derived from the common sequence of the ZIKV E protein (EZIKV). The immunogenicity of this vaccine was assessed in BALB/c mice, and it was discovered that it triggered strong specific-EZIKV immune responses in terms of both humoral and cellular aspects, suggesting that it is a safe and suitable choice for preventing ZIKV infection. Compared to traditional vaccines, this vaccine offers striking safety and can be designed and produced efficiently with high purity. Despite being a vaccine, its low ability to trigger an immune response necessitates the use of more powerful doses and adjuvants to achieve an effective level of protection (Table 2) (Amaral et al. 2020). To overcome this problem, coexpressing prM/E proteins in insect cells or yeast cells to self-assemble into VLPs has been shown to be an effective strategy (Liu et al. 2014, Suphatrakul et al. 2015). VLP-based vaccines can induce the needed immune response and can be readily and cost-effectively manufactured on a large scale.

Furthermore, VLPs are safe because they do not include any viral genetic material and are thus noninfectious (Table 3) (Amaral et al. 2020). Garg et al. tested the immune response in BALB/c mice vaccinated with ZIKV VLPs generated using a prM-E or C-prM-E (capsid-premembrane-envelope) construct, and reported that C-prM-E VLPs could generate higher neutralizing antibody titers than prM-E VLPs, which suggested that the inclusion of C may be beneficial for ZIKV and other flaviviral VLP vaccines. In addition, they also found that VLP vaccines demonstrate better effectiveness than DNA vaccines in terms of inducing a neutralizing antibody response. Furthermore, they developed a multivalent vaccine targeting chikungunya virus (CHIKV), Japanese encephalitis virus (JEV), YFV and ZIKV termed the CJaYZ vaccine based on VLPs and tested the immunogenicity of the multivalent, bivalent and tetravalent vaccines in a murine model. High levels of neutralizing antibodies were detected in all groups of immunized mice. Among these, the titers of monovalent groups were the highest because they may receive the highest antigen dose. Mice that received bivalent and tetravalent vaccine combinations displayed a minor decrease in neutralizing antibody titers, which can be attributed to the reduction in antigen dose administered (Garg et al. 2017, 2020). Boigard et al. developed ZIKV VLPs by coexpressing C-prM-E and NS2B/NS3 proteins and tested their effectiveness against ZIKV in BALB/c mice. Compared with the inactivated ZIKV vaccine, these ZIKV VLPs could stimulate prominently greater neutralizing antibody titers, which indicated that chemical inactivation may have deleterious effects on neutralizing epitopes within the E protein. Moreover, ZIKV VLP vaccination elicited strong neutralizing antibody responses and hardly mediated ADE in response to DENV-2, which may significantly contribute to protection against ZIKV infection. The lack of infectivity of ZIKV VLPs eliminates the need for chemical inactivation, which may influence the efficacy and safety of vaccines (Boigard et al. 2017). Cabral-Miranda et al. developed a vaccine candidate against ZIKV by coupling E-DIII to *cucumber mosaic virus* (CuMVtt) VLPs and formulating a product with a dioleoyl phosphatidylserine (DOPS) adjuvant. This vaccine could induce antibodies efficiently and neutralize the virus without predisposing the recipient to ADE with DENV infection (Cabral-Miranda et al. 2019). Currently, vaccines based on VLPs do not contain replicating viral genetic material and typically have excellent safety profiles. Moreover, substantial improvements in VLP production and adjuvant optimization can lead to the licensing of several VLP-based vaccines to prevent infectious diseases, including influenza and hepatitis A (Bovier 2008, Herzog et al. 2009, Cimica and Galarza 2017).

**Table 3.** ZIKV-VLP-based vaccines in the developmental stage.

Type of VLP	Immunogen	Adjuvant tested	Expression system	Assembly constructs	Merits
Chimeric	EDIII	Aluminum and poly (I:C)	*Nicotiana benthamiana*	HBcAg chimera	Induction of neutralizing antibodies and no ADE in vitro.
	EDIII	DOPS	Bacteria cells	CuMVtt VLPs	Induction of neutralizing antibodies and no ADE in vitro.
	PrM/E	Alum	Baculovirus and Sf9 insect cells	C peptide-prM-E	Induction of neutralizing antibodies and T-cell responses.
Zika	PrM/E	TiterMax Gold	Mammalian cells 293T	C-prM-E and NS2B/NS3 (WNV)	Higher level of neutralizing antibodies than with prM-E ZIKV-VLPs and DNA-based vaccines.
	PrM/E	AddaVax	Mammalian cells Expi293	C-prM-E and NS2B/NS3	Higher level of neutralizing antibodies than with a formalin inactivated ZIKV vaccine and no ADE in vitro.

Abbreviations: DOPS, dioleoyl phosphatidylserine; CuMVtt, Cucumber mosaic virus; HBcAg, hepatitis B core antigen.

## Challenges and future perspectives

Many researchers have attempted in recent years to create vaccines that are both effective and safe for the prevention of ZIKV infection. Various vaccine candidates have undergone preclinical testing and have shown potential for further advancement. Currently, there are various phase I clinical trials underway for five types of vaccines against ZIKV, two of which are in the phase II stage. However, several challenges are being faced in vaccine development regarding safety and efficacy that need to be considered before these vaccines are licensed and used in the clinic. The first concern is the need to establish well-characterized pregnancy models of ZIKV infection that are relevant to human disease and congenital Zika syndrome (CZS) prevention. Mice are often used to model human disease due to their affordability and ease of breeding and manipulation. Nevertheless, their gestation period is brief, and they possess a placental structure that is fundamentally different from that of NHPs and humans (Schmidt et al. 2015, Wahid et al. 2017). Moreover, the neonatal Fc receptor for IgG (FcRn) facilitates dissimilar antibody transfer across the placenta between mice and nonhuman primates(FcRn) (Roopenian and Akilesh 2007). NHPs are more vulnerable to ZIKV infection than model mice and exhibit placental architectures and pregnancies similar to those of humans, making them a better model. Additionally, immune responses to ZIKV infection in pregnant baboons and rhesus macaques have been shown to be strong (Kim et al. 2019). Therefore, it is important to acknowledge the impact of existing flavivirus immunity on vaccine safety, efficacy, and immunogenicity during pregnancy. Studies have shown that the presence of neutralizing antibodies significantly contributes to protection against ZIKV infection, but evaluating the efficacy of vaccines for preventing CZS during pregnancy is imperative. It is crucial to note that vaccines may not be entirely safe, nor do they impart lifelong immunity to all recipients, and challenges associated with vaccination during pregnancy, such as the immunization of vaccine platforms with new technologies, must be tested in humans (Kim et al. 2019). The safety and efficacy of vaccines for pregnant women and other high-risk individuals must be considered in the design process, given the clinical symptoms caused by ZIKV infection. Furthermore, as IgG elicited by vaccines and flaviviruses can be transferred through the placenta, it is crucial to consider how these antibodies will affect the health of fetuses and newborns during vaccine development. Since ZIKV and DENV share antigens, immunization with a ZIKV vaccine may cause cross-reactive antibodies to worsen several symptoms of DENV infection, especially in first-ever vaccine recipients (Katzelnick et al. 2020, Röbl-Mathieu et al. 2021). Furthermore, it is unclear how having immunity to other flaviviruses may affect a person's susceptibility to ZIKV infection, including the duration of immunity following ZIKV infection. To advance the development of vaccines for congenital and perinatal infections and safeguard newborns, researchers must perform further trials in the context of mother-to-child transmission. These factors must also account for the impact of pregnancy on immunity as well as the timing of disease detection and screening (Singh et al. 2020). Due to the success of the coronavirus disease 2019 (COVID-19) vaccination in pregnant women, it is important to closely monitor the impact of ZIKV vaccination on pregnant women. This approach entails enhancing surveillance for potential neurological and autoimmune complications and identifying any adverse reactions caused by vaccination to guarantee the health of pregnant women. Moreover, additional time is needed to demonstrate the efficacy of vaccines in a combined population of pregnant and nonpregnant individuals (Rid and Miller 2016, Dean et al. 2019, Male 2022).

Vaccine research and development heavily rely on animal models and observational cohorts in epidemic areas, making it impossible to estimate efficacy without ongoing transmission. Although global cases of ZIKV disease have declined since 2017, in various countries and endemic regions, transmission continues to occur at low levels, presenting challenges for vaccine development and evaluation. To address this, it is necessary to develop controlled human infection models for vaccine efficacy testing and to conduct further clinical experiments targeting regions severely affected worldwide (Pattnaik et al. 2020). Due to the significant impact of ZIKV on pregnant women, various strategies, such as enrolling only females who are not of child-bearing age or require the use of highly effective contraception during the study period, have been suggested to minimize the risk of infection in nonparticipants (Durbin and Whitehead 2017). Innovative designs and ethical considerations are also vital in assessing vaccine safety and efficacy during an outbreak (Rid and Miller 2016, Dean et al. 2019).

## Conclusions

In general, five types of vaccines against ZIKV are currently undergoing clinical trials: live-attenuated vaccines, inactivated vaccines, nucleic acid vaccines, viral vectored vaccines, and recombinant subunit vaccines. Inactivated vaccines offer convenient production, safety, and preservation advantages; however, their immunogenicity is generally lower than that of live attenuated vaccines, and adjuvants are needed to stimulate high-level immune responses. DNA vaccines offer stability and mass production advantages, but their low immunogenicity must be addressed. To enhance their effectiveness, vaccination strategies involving priming and boosting with different vaccine combinations have proven successful in animal models. Compared to inactivated vaccines, viral vectored vaccines do not require adjuvants or booster immunizations. Recombinant subunit vaccines stimulate long-lasting protective and therapeutic immune responses and are currently a practical and feasible way to develop immunity against ZIKV.

Furthermore, developing safe and effective ZIKV vaccines remains challenging for researchers and scientists. To market and promote awareness, significant steps such as mass production, adjuvant selection, optimal animal model establishment, patient recruitment, transportation methods, and immunization schemes are necessary. Therefore, governments should increase their efforts to assist companies in terms of policies and funds to perform further clinical trials and manufacture ZIKV vaccines for deployment.

## Institutional review board statement

Not applicable.

## Informed consent statement

Not applicable.

## Data Availability

Not applicable.
